# Intrapericardial rupture of right atrial angiosarcoma without cardiac tamponade

**DOI:** 10.1093/ehjcr/ytaf154

**Published:** 2025-03-27

**Authors:** Tasuku Sato, Shohei Moriyama, Mitsuhiro Fukata, Akira Shiose

**Affiliations:** Heart Center, Kyushu University Hospital, 3-1-1 Maidashi, Higashi-ku, Fukuoka 812-8582, Japan; Department of Hematology, Oncology, and Cardiovascular Medicine, Kyushu University Hospital, 3-1-1 Maidashi, Higashi-ku, Fukuoka 812-8582, Japan; Department of Hematology, Oncology, and Cardiovascular Medicine, Kyushu University Hospital, 3-1-1 Maidashi, Higashi-ku, Fukuoka 812-8582, Japan; Department of Cardiovascular Surgery, Kyushu University Hospital, 3-1-1 Maidashi, Higashi-ku, Fukuoka 812-8582, Japan

## Case description

**Figure 1:**
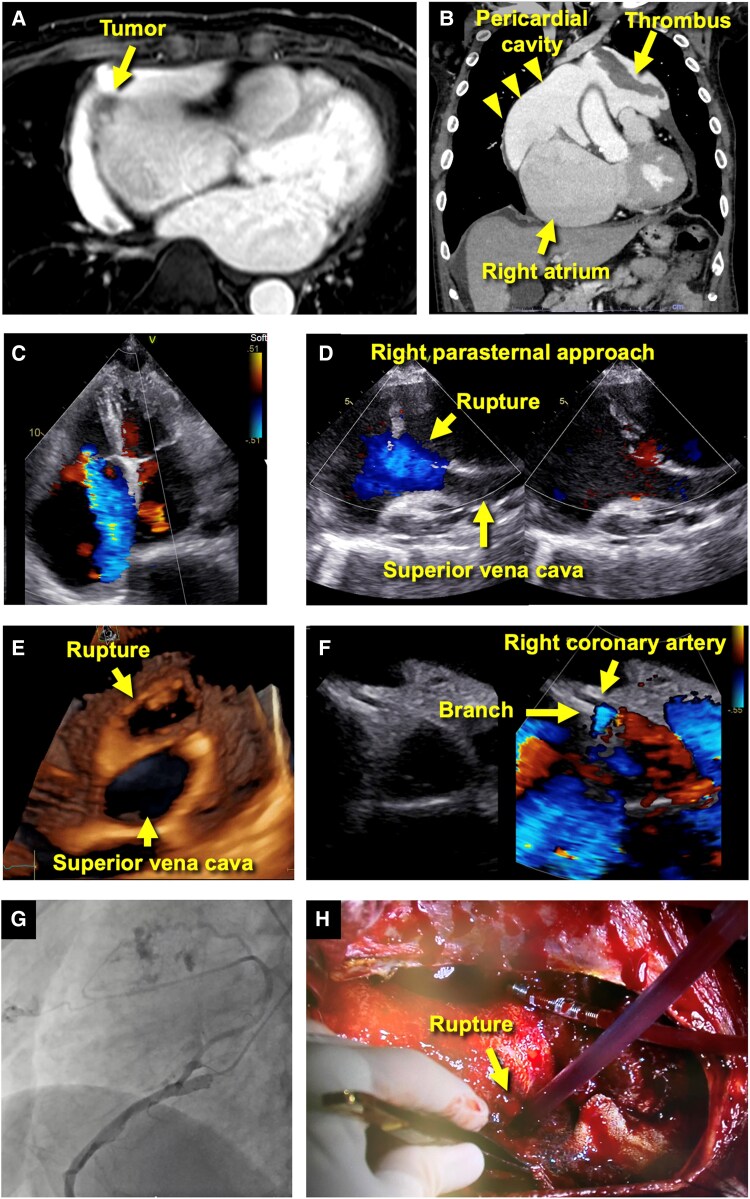
(*A* and *B*) Computed tomography (axial and coronal views) revealed a rupture in the right atrium along with pericardial effusion containing thrombus. (*C* and *D*) Transthoracic echocardiography in the apical four-chamber view failed to visualize the rupture but was identified in the right parasternal view. (*E*) Three-dimensional transthoracic echocardiography (right parasternal view) reconstructed an *en face* view of the rupture and a cross-section of the superior vena cava. (*F*) Parasternal short-axis view with colour Doppler detected high-flow signals in a branch of the right coronary artery, suggesting a fistula to the pericardial space. (*G*) Coronary angiography confirmed the presence of the fistula. (*H*) Surgery revealed the right atrial rupture, and the atrial wall was reconstructed.

A 74-year-old man presented with shortness of breath. He had a history of persistent atrial fibrillation and past smoking but had no other cardiovascular risk factors or family history of cardiovascular or malignant disease. Computed tomography (CT) revealed a cardiac tumour in the right atrium (RA) free wall myocardium, accompanied by a RA-pericardial fistula near the mass and significant pericardial effusion extending cranially (*Figure 1A* and *B*). Multiple pulmonary masses and a mass at the left ventricular apex were also present. These findings suggested a cardiac tumour with distant metastasis and RA rupture, posing a high risk of surgical complications. Consequently, best supportive care (BSC) was initially chosen. However, follow-up CT one month later showed regression of the pulmonary metastases and the RA tumour, leading to the withdrawal of BSC and prompting referral to our hospital for multidisciplinary management.

Transthoracic echocardiography demonstrated marked RA enlargement and severe tricuspid regurgitation (*Figure 1C*). Notably, there was no evidence of heart chamber collapse indicative of cardiac tamponade. A right parasternal approach revealed a defect (2 × 3 cm) near the superior vena cava, with bidirectional blood flow on colour Doppler (*Figure 1D* and *E*, Supplementary data online, *Video S1*). Continuous blood flow from a branch of the right coronary artery into the pericardial space was identified (*Figure 1F*). Coronary angiography confirmed the presence of a feeding artery entering the pericardium (*Figure 1G*, Supplementary data online, *Video S2*). This atypical presentation of RA rupture without cardiac tamponade can be attributed to gradual pericardial adaptation facilitated by the tumour’s neovascularization, bidirectional flow between the RA and pericardium, and localized pericardial effusion. The patient underwent resection of the RA and left ventricular tumours, tricuspid valve repair, and RA wall reconstruction (*Figure 1H*). Due to a bleeding tendency, bi-stage haemostasis was required; however, no serious complications occurred. Histopathological analysis confirmed the diagnosis of angiosarcoma, and weekly paclitaxel therapy was initiated. One year later, the patient remains on chemotherapy.

Cardiac angiosarcoma most commonly originates in the RA and is associated with a poor prognosis. It can lead to severe complications, including myocardial rupture, pericardial effusion, and cardiac tamponade.^1–3^ This case highlights the exceptional rarity of RA rupture without tamponade and underscores the crucial role of echocardiography and CT in its accurate assessment and effective management.

## Supplementary Material

ytaf154_Supplementary_Data

## Data Availability

All data are incorporated into the article and its online Supplementary material.

## Notes

**Funding:** This work was supported by clinical trial funding from ALEXION (GAJC202043).
