# Supplementary material for: Intrapericardial rupture of right atrial angiosarcoma without cardiac tamponade
Source: Eur Heart J Case Rep. 2025 Mar 27;9(4):ytaf154. doi: 10.1093/ehjcr/ytaf154 (PMC11997428; doi:10.1093/ehjcr/ytaf154)
Supplement: ytaf154_Supplementary_Data [file ytaf154_supplementary_data.zip › Supplemental data (figure legend).docx]

**Supplemental data**

**Supplemental Movie 1 (*cf.* Panel D and E in Figure)**

A right parasternal approach revealed a rupture in the right atrial wall below the superior vena cava, with a large communication to the pericardial cavity. Color Doppler demonstrated bidirectional flow. At the margin of the rupture, a narrow inflow signal was observed, suggesting the presence of a coronary artery fistula. Three-dimensional transthoracic echocardiography revealed a communication measuring 2 cm × 3 cm.

**Supplemental Movie 2 (*cf.* Panel G in Figure)**

Coronary angiography showed a branch originating from the proximal right coronary artery, flowing into the pericardial space through a perforation in the right atrium.
